# Microbiota-associated molecular genotoxicity

**DOI:** 10.18632/aging.203446

**Published:** 2021-08-18

**Authors:** Irene Maier

**Affiliations:** 1 Department of Environmental Health Sciences, University of California, Los Angeles, CA 90095, USA; 2 Present address: Department of General Surgery, Medical University of Vienna, Vienna, Austria

**Keywords:** radiation, microbiota, bacterial indicator phylotypes, bone loss, microstructure, interleukin-17

The composition of the intestinal microbiota represents an early indicator of chronic post-radiation outcomes in elderly bone tissue, since bacterial indicator phylotypes have become a novel biomarker for radiation dosimetry in human body. Microbiota influence therapeutic approaches and, rather than cervical viral loads, correlate with clinical outcomes in pelvic radiotherapy [1]. I hypothesize that intestinal microbiota induce aged alterations in DNA repair in a sex-specific manner, impacting bone microstructure and osteoblast dysfunction in irradiated mice.

In a recent study, we performed analysis of the fecal bacteria in relation to bone quality concerning skeletal bone structure, body weight and relative bone volume, showing reduced tibia cortical thickness in silicon ion irradiated mice [2]. Analyses between the gut microbiota and bone microstructure revealed *Bacteroides massiliensis, Muribaculum sp.,* or *Prevotella denticola* were different between conventional microbiota and anti-inflammatory microbiota (edgeR, FDR-P<0.05), which was found conditional on mucosa-associated dysbiosis under both disturbances of interleukin (IL)-17 signaling and exposure to radiation alone.

Restricted bacterial compositions (restricted microbiota) indicated that gut bacteria interactions were potentially involved in the differentiation of naive T cells into IL-17 producing T-helper (Th) cells. *Turicibacter sp.,* for example, was directly correlated with trabecular separation in cancellous bone measured in female proximal tibiae in anti-IL-17 treated mice (edgeR, FDR-P=0.048, unpublished data). Previously, *Turicibacter* was found to be one of the phylotypes in female colons that increased higher in relative abundance in restricted microbiota than conventional microbiota due to oral administration of *Lactobacillus johnsonii* (LBJ) suspension. The abundance of *Turicibacter sp*. in the small intestine correlated with radiation-induced genotoxicity (P=0.0285) [2]. Growing body fat in LBJ-inoculated and irradiated adult mice was to a greater extent correlated with radiation resistance of villus epithelial cells and enhanced expression of fasting-induced adipose factor Fiaf in blood [2, 3] (a key mediator of the microbiota's ability to promote host storage of energy that is also known as angiopoietin-like 4) [3]. Among the 20 highest differentially abundant taxa, very few changed due to IR, including an unclassified *Bacteroidetes* [2].

While persistent DNA damage diversely impacts and alters functional human mesenchymal stem features, IR aggravates osteogenic differentiation dysfunction and the expression of senescence markers independent of tumor suppressor gene p53. More specifically, hematopoietic cell senescence and up-to-a-year delayed intestinal epithelial cell migration have been associated with high charge and energy (HZE) particle beam radiation [4]. Post-radiation restricted microbiota were characteristic of the depletion of pathogens from the gut of ataxia-telangiectasia-mutated (Atm)-deficient mice. Adult mice showed spontaneous leukocyte genotoxicity with aging, resulting in exaggerated levels of genomic instability and peripheral DNA lesions in wild-type and those mice lacking the DNA damage sensor protein Atm for the repair of double-stranded DNA breaks. When female mice were injected with neutralizing anti-IL-17 antibodies pre- and post-IR and irradiated, however, low levels of interferon (IFN)-γ in the small intestine were associated with higher radiation susceptibility in females in an immune-regulatory context [2], followed by Th17 cell migration into the bone marrow [5]. Independent of radiation-induced genotoxicity and cytokine expression in the gut, the intestinal microbiota composition revealed a potential antagonist for the pathogenicity of Th17 cells and activation of regulatory T cells, mediated by transforming growth factor (TGF)-β and IL-1β [2, 5]. Blocking IL-1 showed that IL-1 was a major driver of tissue damage, as well as microbiota were associated with enhanced expression of tumor necrosis factor (TNF)-α [6], particularly in irradiated bone marrow [2]. We associated *Muribaculum intestinale* with adopted radiation-resistance in murine colons. Quinn L and coworkers [7] demonstrated the loss of menaquinone-producing microbes along with a reduction of *M. intestinale,* among others, due to antibiotic treatment, was caused by its decreasing canonical biosynthesis genes in gastric cancer therapy. Thus, *Helicobacter pylori* eradication altered enteric vitamin K production and diminished carboxylated osteocalcin.

Together, cancer is an age-related disease that is mediated by the microbiota as it promotes an apoptotic but anti-inflammatory cancer barrier. Transplants of irradiated microbiota, however, predisposed germ-free recipient mice for radiation injury and colitis. Radiation treatment, i.e. fractionated radiotherapy applying high dose of ionizing radiation (>50 Gy), induced localized microbial dysbiosis (microbiota signatures). Shifts in intestinal microbiota differed in luminal microbiota and bacterial composition in irradiated feces compared with non-irradiated gut segments [6]; until today, the effects of gut microbiota on aging, intervention and treatments of aging-related diseases were deeply investigated in the small-animal models of vertebrae. Yu M, et al. confirmed the functional relevance of TNF for the bone catabolic activity of parathyroid hormone and showed that a low-calcium diet can lead to bone resorption, high bone turnover, and impaired bone trabecular micro-architecture in bones [5], such as the hard palate, mandible, vertebrae, femur, and proximal tibia [5, 8].

Collectively, our research provides linkages between gut bacteria and radiation-induced bone loss, imparting the upregulation of bacterial indicator phylotypes in the irradiated intestinal microbiota to modulate bone health in prostate and colorectal cancer patients. Preclinical data are summarized for the manipulation of the intestinal microbiota to enable prevention of osteoporosis. Moreover, several issues beyond the current risks in spaceflight are discussed to elucidate how the microbiome may reduce symptoms of aging, osteoclastogenesis and cell death in astronauts when traveling to Mars.
